# Label-free quantitative proteomic analysis of molting-related proteins of *Trichinella spiralis* intestinal infective larvae

**DOI:** 10.1186/s13567-019-0689-0

**Published:** 2019-09-23

**Authors:** Hua Nan Ren, Ruo Dan Liu, Yan Yan Song, Tong Xu Zhuo, Kai Xia Guo, Yao Zhang, Peng Jiang, Zhong Quan Wang, Jing Cui

**Affiliations:** Department of Parasitology, Medical College, Zhengzhou University, Zhengzhou, 450052 China

## Abstract

Molting is a key step for body-size expansion and environmental adaptation of parasitic nematodes, and it is extremely important for *Trichinella spiralis* growth and development, but the molting mechanism is not fully understood. In this work, label-free LC–MS/MS was used to determine the proteome differences between *T. spiralis* muscle larvae (ML) at the encapsulated stage and intestinal infective larvae (IIL) at the molting stage. The results showed that a total of 2885 *T. spiralis* proteins were identified, 323 of which were differentially expressed. These proteins were involved in cuticle structural elements, regulation of cuticle synthesis, remodeling and degradation, and hormonal regulation of molting. These differential proteins were also involved in diverse intracellular pathways, such as fatty acid biosynthesis, arachidonic acid metabolism, and mucin type *O*-glycan biosynthesis. qPCR results showed that five *T. spiralis* genes (cuticle collagen 14, putative DOMON domain-containing protein, glutamine synthetase, cathepsin F and NADP-dependent isocitrate dehydrogenase) had significantly higher transcriptional levels in 10 h IIL than ML (*P* < 0.05), which were similar to their protein expression levels, suggesting that they might be *T. spiralis* molting-related genes. Identification and characterization of *T. spiralis* molting-related proteins will be helpful for developing vaccines and new drugs against the early enteral stage of *T. spiralis*.

## Introduction

*Trichinella* is an obligate parasitic nematode of animals worldwide. Human *T. spiralis* infection primarily results from eating raw or semiraw animal meat infected with *Trichinella spiralis* muscle larvae (ML). Trichinellosis outbreaks were reported in 55 countries around the world, resulting in 65 818 cases and 42 deaths, from 1986 to 2009 [1]. In Chinese Mainland, 14 of 15 trichinellosis outbreaks were due to infected pork during 2004–2009, and pork is the predominant infection source [2, 3]. *Trichinella* is not only a public health harm but also a serious hazardous to animal food safety. Vaccine development is needed to eliminate the transmission of *Trichinella* among domestic animals [4–6].

After being eaten, ML encapsulated in animal meat emerges in the host’s stomach and develops into its first stage, called intestinal infective larvae (IIL1), at 0.9 hours post-infection (hpi). The IIL1 penetrate the intestinal epithelium and undergo the first molting to develop into IIL2 at 10 hpi. They subsequently molt three times into IIL2–IIL4 to develop into the adult worm (AW) within 10–30 hpi [7–9]. A cuticle covers the surface of the *Trichinella* worm body, and molting is the basis of *Trichinella* growth and development, which occurs in the development of all members of the nematode. The presence of various enzymes in cuticles of *T. spiralis* IIL suggests that the cuticles may contain the molting-related enzymes [10]. Each molting consists of the new cuticle formatting, old cuticle loosening and rupturing, and larval escape from previous-stage larval cuticle [11]. Molting is controlled by ex-sheathing fluid secreted by the larvae; however, which kinds of proteins in ex-sheathing fluid participate in the shedding of old cuticle and the formation of neocuticle are not yet understood. Hence, agents that inhibit or block molting have been a focus for developing new drugs or vaccines against nematode infection. In addition, studies on molting will be valuable to understand the growth and developmental processes of nematodes [12].

Molting is an important strategy for *T. spiralis* to adapt to the environmental intestinal milieu. Previous studies revealed that *T. spiralis* infective larvae molted and developed to the adult stage after they were inoculated onto intestinal epithelial cell monolayers cultured in vitro. Approximately 50% of IIL larvae molted when they were cultivated in an epithelial cell monolayer for 5–9 days [13]. If the old cuticle was not completely shed, the larvae were still enclosed in the sheath and could not develop into the adult stages [14]. However, the biological processes that regulate *T. spiralis* molting are not fully clear.

The encapsulated-stage ML lodge in the nurse cells of the host’s skeletal muscle, and their surrounding environment is relatively stable. The ML may live from 1 to 2 years to 10–15 years without any major harm [15]. Hence, ML has been used as a normal control for *T. spiralis* protein expression. Following ingestion, *T. spiralis* ML are liberated from their capsules in the host’s stomach and activated into IIL1 by exposure to enteral content or bile at 0.9 hpi. After they invade the host’s intestinal epithelium, the IIL completes four molts from IIL1 to IIL4 at approximately 10, 10–14, 15–22 and 23–30 hpi, respectively [16]. The 10 h IIL might express multiple molting-related proteins that are involved in new cuticle generation, old cuticle loosening and ex-sheathing for the first molting of this parasitic nematode. Furthermore, since the enteral-stage IIL and AW parasitize the complicated intestinal environment, the IIL at 10–30 hpi and AW possibly express various proteins that participate in other processes of *Trichinella* development, such as digestion, immune escape, detoxification, reproduction, and so on [6, 17–19]. To screen and identify the *T. spiralis* molting-related proteins as far as possible, 10 h IIL were selected for quantitative proteomic analysis. In this study, the proteomic profile variation between *T. spiralis* ML and 10 h IIL was determined using label-free quantitative proteomic technology with bioinformatics, which is expected to provide new insight to guide the study of *T. spiralis* larval molting, growth and development [20].

## Materials and methods

### Worm and mice

*Trichinella spiralis* strain (ISS534) from a domestic pig in central China was kept by mouse serial passaging in our department [21]. Specific-pathogen-free (SPF) 8-week-old female mice were obtained from the Experimental Animal Center of our university. The animal experiment protocol was approved by the Life Science Ethics Committee of Zhengzhou University (No. SCXK 2017–0001).

### Intestinal infective larvae at different times post-infection

The MLs were obtained from artificially digesting *T. spiralis*-infected mouse muscles with 1% pepsin [22, 23]. Each mouse was orally inoculated with 5000 ML, and IIL were isolated from the small intestine at 8, 10, 12, 14, 16, and 18 hpi [24]. The IIL molting process was observed and counted under microscopy. Fifty IIL were randomly selected from each time point, and three replicates were observed. All larvae were washed thoroughly with sterile PBS and then stored in liquid nitrogen.

### Protein extraction

The ML and 10 h IIL were homogenized in a tissue grinder. The larval fragment was further homogenized by ultrasonication as reported [25]. The homogenate was boiled for 15 min. Following centrifugation at 14 000 *g* for 40 min, the supernatant was filtered using 0.22 µm filters. The protein concentration was determined using a BCA kit (Bio-Rad, USA).

### Protein digestion

Approximately 200 μg of larval proteins was added to 30 μL of SDT buffer (150 mM Tris–HCl, 4% SDS and 100 mM DTT) and filtered with UA buffer (8 M urea, 150 mM Tris–HCl). To inhibit the cysteine residues, larval proteins were incubated with 100 μL iodoacetamide for 30 min in darkness. After washing, the protein sample was digested with 4 μg trypsin at 37 °C overnight, and the resulting peptides were recovered, desalted and concentrated as described [26]. The contents of peptides were determined using a UV light spectral density at 280 nm.

### LC–MS/MS and protein datum analysis

LC–MS/MS was carried out using a Q Exactive mass spectrometer (Thermo Fisher Scientific, USA) as reported [27]. The MS data were analyzed by MaxQuant software (version 1.5.3.17) against the *Trichinella* data from the UniProt database. The differentially expressed *T. spiralis* proteins between the ML and 10 h IIL stages were those with a 2.0-fold change compared with one another [12]. Molecular pathway analyses through Gene Ontology (GO) and Kyoto Encyclopedia of Genes and Genomes (KEGG) were conducted to analyze the possible enrichment of differentially expressed proteins with specific biological features. The mapping of protein interactions was performed by statistical analysis at a low confidence score in the STRING database [27].

### qPCR

Eight genes from differently expressed *T. spiralis* proteins were selected to investigate their transcription levels using qPCR [9]. Total RNA of ML and 10 h IIL was extracted with Trizol reagent (Takara, Japan) and transcribed into cDNA as templates for qPCR. The gene-specific primers were designed using Primer 5.0 software (Table 1) and synthesized by Sangon Biotechnology Co. (Shanghai, China). The *T. spiralis* GAPDH gene (GenBank: AF452239) was also amplified as an internal control gene [28]. The gene transcription level was evaluated using the comparative Ct (2^−ΔΔCt^) method as described [29, 30]. Each sample was run in triplicate.

**Table 1:** **Primers used in the quantitative real-time PCR assays**

Gene description	NCBI accession	Primer sequence	Product size (bp)
Cuticle collagen 14 (E5RZT0)	XM_003381945.1	F 5′-TCTGGTGATGCATCGGATCG-3′ R 5′-TCCTTCAGCACACGCTTCAT-3′	113
Cytochrome P450 4V2 (E5SY27)	XM_003370180.1	F 5′-CGCAAACTGCTCACACCATC-3′ R 5′-CGCAGATGATGTCCAAAGCG-3′	172
Endoplasmic oxidoreductin-1 (E5SKW7)	XM_003373203.1	F 5′-AGGTAATGCGAACAAGGCGA-3′ R 5′-ATGGGTCTGCAACTTTCCCC-3′	124
Putative DOMON domain-containing protein (E5SFI2)	XM_003378229.1	F 5′-GCTGATTCCGCTTCTCCAGT-3′ R 5′-CGGGTCTGGTTTTTCGCTTG-3′	157
Glutamine synthetase (E5SIC2)	XM_003374954.1	F 5′-CCCGTTTCGACTGGGAAAGA-3′ R 5′-CGAGATCGAGCAGCGTGTAT-3′	167
RNA-binding protein 47 (E5S1I0)	XM_003378983.1	F 5′-CGCAATACGCGTCCAAGAAG-3′ R 5′-AGAGACCGATGATGGTGGGA-3′	174
Cathepsin F (E5SFB3)	XM_003378197.1	F 5′-ATGGCCCCACAATTTTTGCC-3′ R 5′-TCCATTCGCTCGCTGATGTT-3′	133
Isocitrate dehydrogenase, NADP-dependent (E5S5M3)	XM_003380431.1	F 5′-TGTACTCATGTGCCCGGATG-3′ R 5′-GGGGTTCGTTTCGATCCAGT-3′	176
GAPDH (Reference)	AF452239	F 5′-GATGCTCCTATGTTGGTTATGGG-3′ R 5′-GTCTTTTGGGTTGCCGTTGTAG-3′	196

### Statistical analysis

The data were analyzed with SPSS for Windows (version 20.0, SPSS Inc., Chicago, IL, USA). Relative gene expression levels in ML and 10 h IIL stages are shown as the mean ± standard deviation (SD), and the differences between two groups were assessed using Student’s *t* test. *P* < 0.05 was regarded as statistically significant.

## Results

### Molting and ecdysis

**Figure 1:**
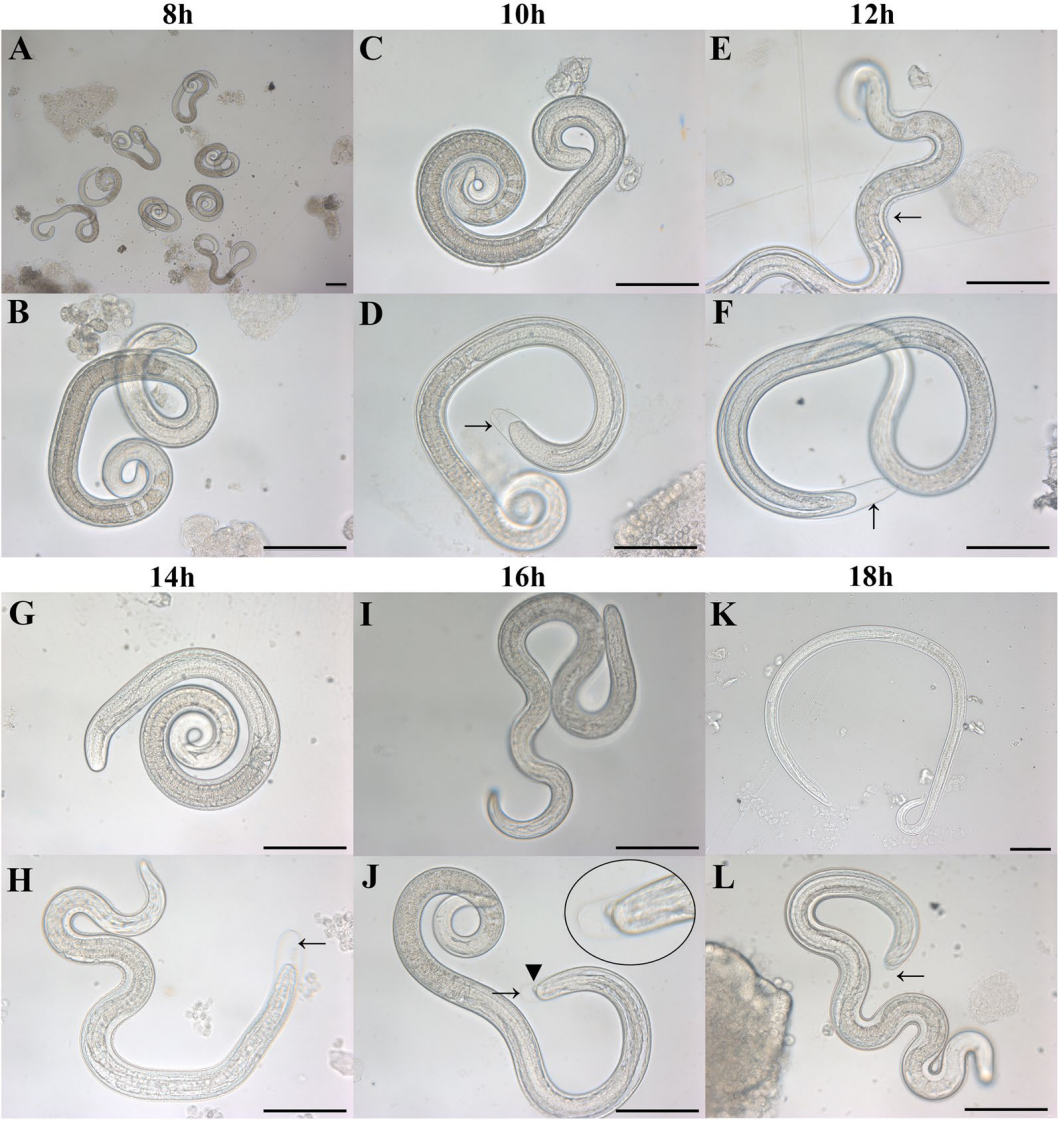
**Molting of** ***T. spiralis*** **IIL larvae at different time points post-infection.** Larval molting was observed at various times post-infection by microscopy. The larvae carrying a sheath at the posterior end were counted as molting worms. **A, B, C, G, I, K**: IIL without molting. **D, E, F, H, J, L**: IIL encased in cuticle (arrow). Copulatory appendage is indicated with a triangle.

**Figure 2:**
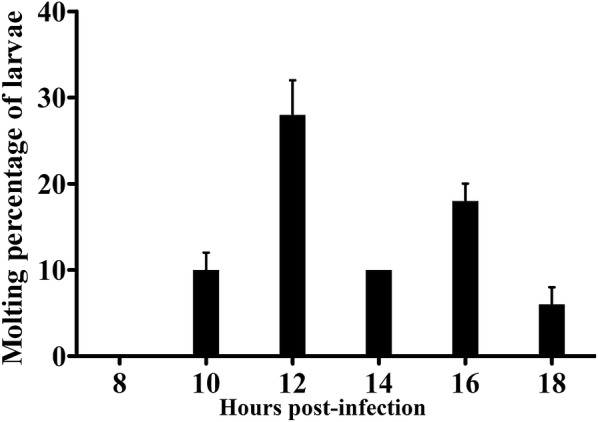
**The percentage of molting larvae at different times post-infection.** *T. spiralis* IIL were collected at different times after infection. One hundred fifty IIL were randomly divided into three groups (50 IIL each group). The molting at 8, 10, 12, 14, 16, and 18 hpi was observed by microscopy. The maximum molting rate was 28%, at 12 hpi.

The molting of *Trichinella* IIL at 8, 10, 12, 14, 16, and 18 hpi is shown in Figure 1. The IIL began to molt at 10 hpi, and the larval molting rate was up to 28% at 12 hpi (Figure 2). The copulatory appendages of the males were clearly observed at 16 hpi under microscopy.

### Protein identification and quantification

**Figure 3:**
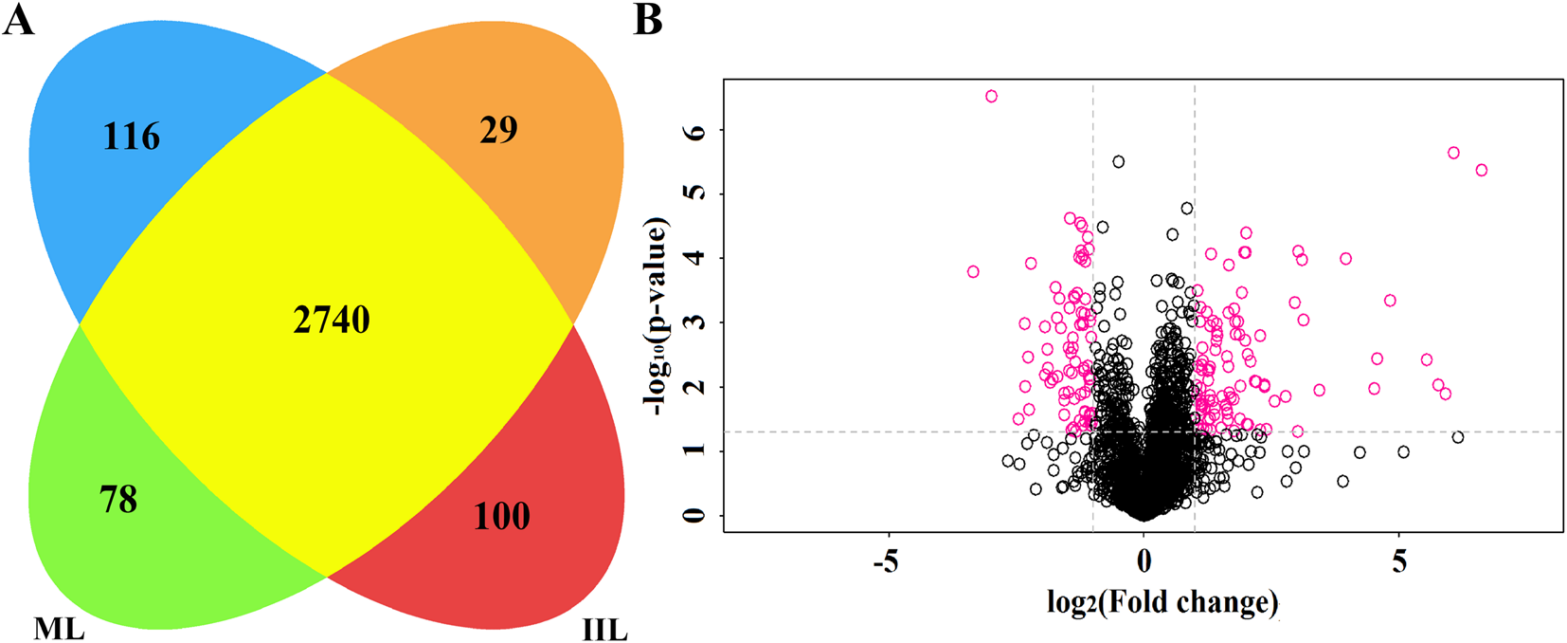
**Results of LC–MS/MS of ML and 10** **h IIL proteins. A** The cartoon showing the intersection of differentially expressed *T. spiralis* proteins between the ML and 10 h IIL. Twenty-nine proteins were found solely in ML (■) and 116 only in 10 h IIL (■). Of the identified proteins, 2562 co-existed in both ML and IIL without a significant difference in expression level (■), 78 were obviously upregulated in the ML (■), and 100 were upregulated in IIL (■). **B** The plot exhibiting the distribution of quantified proteins based on their statistical significance (*P* value) and fold change. The proteins over the dotted line are considered statistically significant (*P* < 0.05), and those beyond the two vertical dotted lines show 2.0-fold changes compared with the ML stage. Differentially expressed proteins between the two larval stages are represented as red dots.

**Figure 4:**
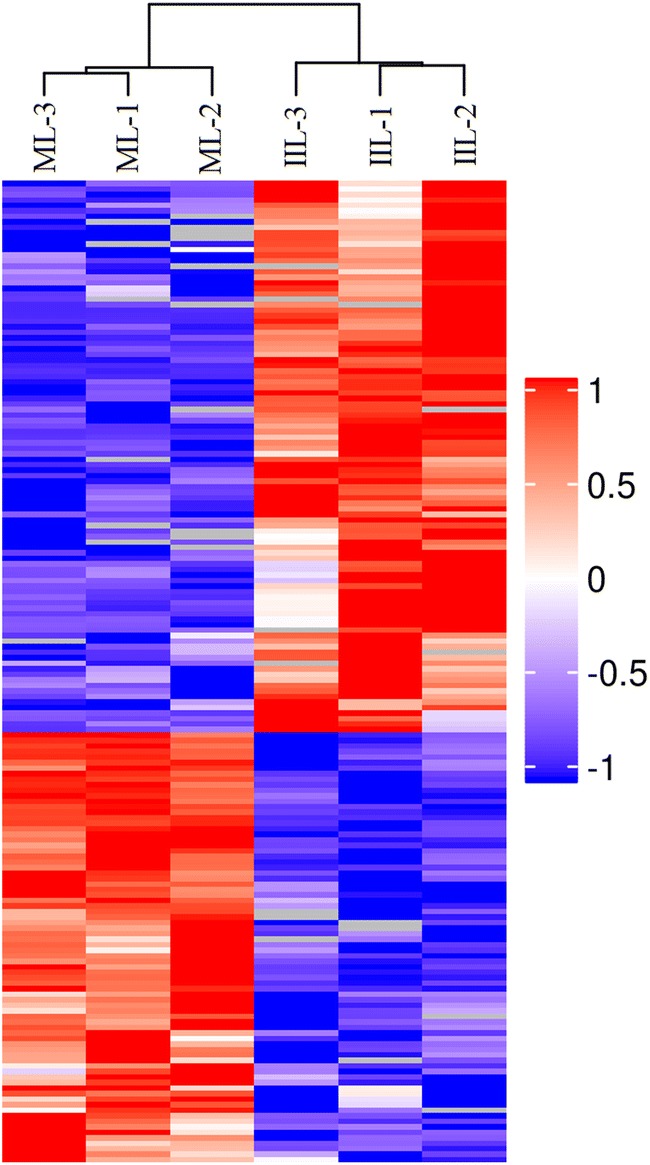
**Heat map of differently accumulated** ***T. spiralis*** **proteins at the ML and 10** **h IIL stages.** These *T. spiralis* proteins were ranked according to their Log2 fold change abundance value. Blue: downregulation; red: upregulation.

The proteomic analysis allowed confident identification of 2885 *T. spiralis* soluble proteins. Of these proteins, 2740 co-existed in both ML and 10 h IIL stages, 29 solely in the ML and 116 only in 10 h IIL. Out of the 2740 co-existing proteins, 100 were significantly upregulated in the 10 h IIL stage (*P* < 0.05), and 78 were significantly downregulated in the 10 h IIL stage (*P* < 0.05) (Figure 3). In this cohort, the downregulation of ADP-ribose pyrophosphatase, serine proteinase, angiotensin-converting enzyme, glycine c-acetyltransferase and carbonic anhydrase was more significant at the ML than at the 10 h IIL stage. At the same time, the upregulation of fatty acid synthase, purine nucleoside phosphorylase, prolyl 4-hydroxylase subunit alpha-2, cuticle collagen 14 was over twofold at the 10 h IIL compared with the ML stage. The significantly upregulated proteins that might be involved in molting are listed in Table 2. A clustering analysis of those 178 proteins common to ML and IIL is shown in Figure 4. A clear set of proteins was significantly downregulated at 10 h IIL, while the majority of IIL larva proteins were upregulated in comparison to the ML stage.

**Table 2:** ***Trichinella spiralis*** **proteins involved in molting that are upregulated in 10** **h IIL**

Protein name	Protein IDs	Gene name	Theor. MW (kDa)	IIL/ML	*P* value	Unique peptides	Coverage (%)	GO annotations
Cuticle structural elements								
Cuticle collagen 14	E5RZT0	Tsp_11095	32.91	23.00	0.0107	5	15.60	CC: collagen trimer; integral component of membrane; MF: structural constituent of cuticle
Putative nematode cuticle collagen N-domain protein	E5SQP6	Tsp_09393	59.89	8.12	0.0496	4	6.30	CC: collagen trimer; integral component of membrane; MF: structural constituent of cuticle
Cuticle collagen rol-6	E5SQP9	Tsp_09396	39.15	–	–	3	13.60	CC: collagen trimer; integral component of membrane; MF: structural constituent of cuticle
Cuticle collagen lon-3	A0A0V1BMR6	lon-3	43.45	–	–	3	12.20	CC: collagen trimer; integral component of membrane; MF: structural constituent of cuticle
Cuticle collagen dpy-7	E5SR67	Tsp_09560	29.37	–	–	6	26.60	CC: collagen trimer
Cuticle collagen 6 Protein roller-8	E5SAG9	rol-8	35.18	–	–	3	9.50	CC: collagen trimer; integral component of membrane; MF: structural constituent of cuticle
Cuticle collagen sqt-1	A0A0V1BL19	sqt-1	34.73	–	–	10	40.00	CC: collagen trimer; integral component of membrane; MF: structural constituent of cuticle
Putative cuticle collagen 2	E5S488	Tsp_03639	26.52	–	–	3	21.10	CC: collagen trimer; integral component of membrane; MF: structural constituent of cuticle
Putative PAN domain protein	E5S7X4	Tsp_07811	67.36	15.61	0.0001	14	36.20	BP: cuticle pattern formation; embryonic ectodermal digestive tract morphogenesis; CC: cytoplasmic side of apical plasma membrane; external side of apical plasma membrane; extracellular space; supramolecular fiber; MF: structural constituent of cuticle
Regulators of cuticle synthesis, maintenance, remodeling and degradation								
Zinc carboxypeptidase superfamily	E5SP83	Tsp_09758	55.37	–	–	5	14.00	MF: metallocarboxypeptidase activity; zinc ion binding
Putative zinc metalloproteinase nas-13	E5S4Y1	Tsp_05948	19.96	–	–	2	11.80	–
Metalloendopeptidase	A0A0V1C237	TFE3	99.70	–	–	16	23.50	BP: protein complex oligomerization; protein maturation by iron-sulfur cluster transfer; MF: metalloendopeptidase activity; zinc ion binding
Cathepsin B	S5M797	–	37.40	2.29	0.0208	2	7.90	MF: cysteine-type peptidase activity
Cathepsin F	E5SFB3	Tsp_02382	41.90	3.26	0.0145	8	25.40	MF: cysteine-type peptidase activity
Attachment components								
Transmembrane cell adhesion receptor mua-3	A0A0V1BV71	mua-3	402.52	3.14	0.0326	1	3.10	CC: collagen-containing extracellular matrix; integral component of membrane; MF: calcium ion binding; extracellular matrix structural constituent
Transmembrane matrix receptor MUP-4	A0A0V1BTR4	mup-4	280.00	2.03	0.0159	49	27.90	CC: collagen-containing extracellular matrix; integral component of membrane; MF: calcium ion binding; extracellular matrix structural constituent
E3 ubiquitin-protein ligase listerin	A0A0V1B0D3	ltn1	201.88	2.34	0.0007	25	19.40	BP: ribosome-associated ubiquitin-dependent protein catabolic process; CC: RQC complex; MF: ligase activity; ubiquitin protein ligase activity
Hormonal regulation of molting								
Cytochrome P450 4V2	E5SY27	Tsp_10837	57.31	8.59	0.0001	22	47.40	CC: integral component of membrane; MF: heme binding; iron ion binding; monooxygenase activity;
Putative nuclear hormone receptor HR3	A0A0V1AYJ5	Hr46	53.37	–	–	1	2.70	CC: nucleus; MF: nuclear receptor activity; sequence-specific DNA binding; steroid hormone receptor activity
Intracellular trafficking components and regulators								
Low-density lipoprotein receptor-related protein	A0A0V1B0U4	lrp-1	74.61	–	–	13	31.00	CC: integral component of membrane; calcium ion binding
Serine/threonine-protein kinase RIO1	A0A0V1BAH6	Riok1	59.34	–	–	3	7.00	MF: ATP binding; protein serine/threonine kinase activity

CC: cellular component, MF: molecular function, BP: biological process.

### Gene Ontology annotation

**Figure 5:**
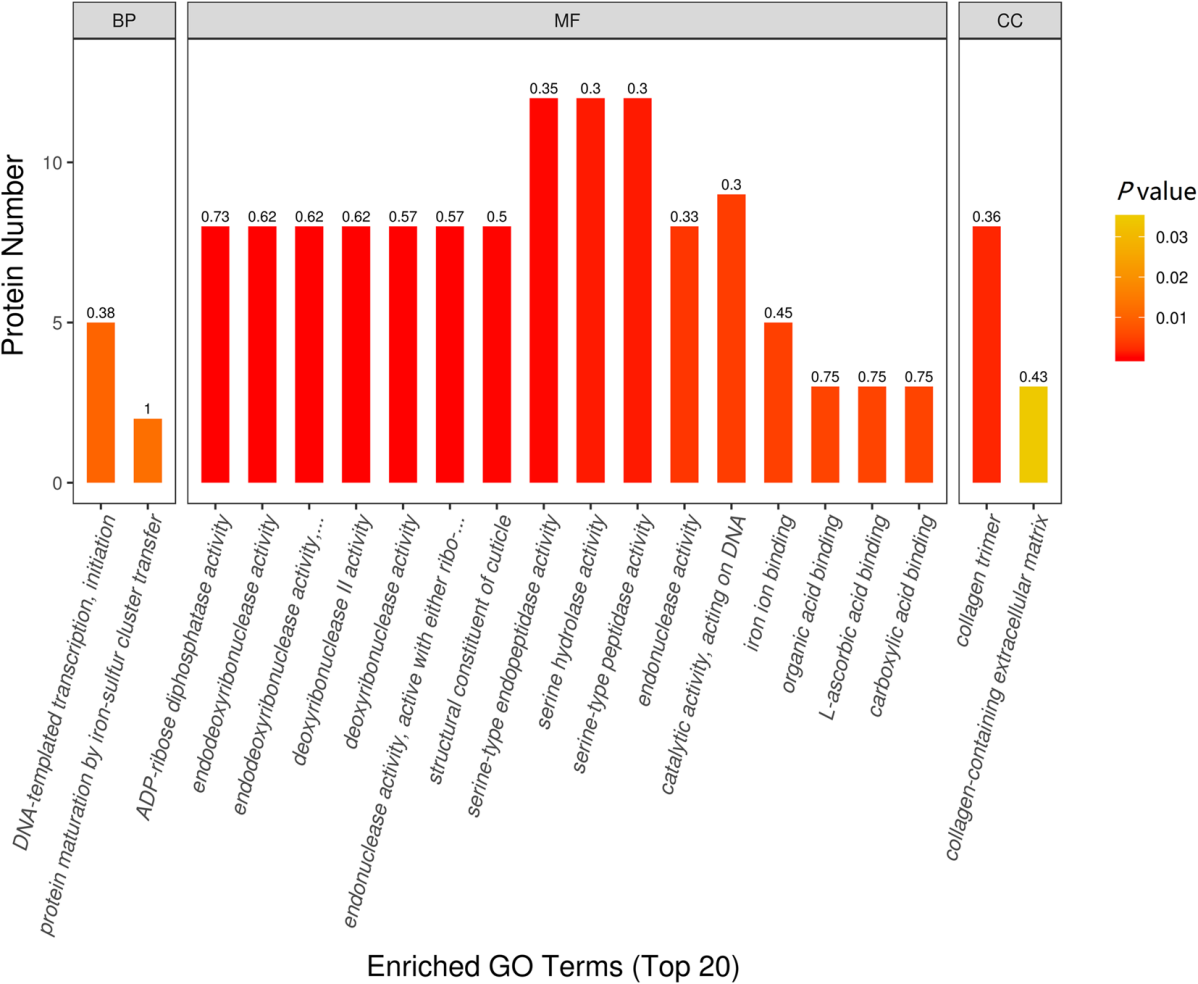
**Enrichment analysis of the 20 most significant GO terms of differentially expressed** ***T. spiralis*** **proteins between ML and 10 h IIL larvae.** The differentially expressed proteins were categorized into biological process, molecular function, and cellular component according to their GO signatures. The number denotes the number of proteins with the given GO annotation.

To determine the biological functions of the differentially expressed *T. spiralis* proteins, the proteins were categorized into three types: biological process, molecular function and cellular components, based on their GO hierarchy. Based on their *P* value, 20 of the most prominent terms for ML and IIL larval proteins are presented in Figure 5. In the molecular function ontology, the majority of protein function is relevant to ADP-ribose diphosphatase activity, endonuclease activity, structural components of cuticle, serine hydrolase activity and catalytic activity. Binding of iron ion, carboxylic acid, L-ascorbic acid and organic acid were the significant and enriched terms of binding activities. Under biological processes, most of the regulated proteins were involved in cellular processes and metabolic processes. DNA-templated transcription, initiation and protein maturation by iron-sulfur cluster transfer were the significant biological processes. There is a close relationship between a protein’s subcellular location and its function. The cellular components collagen trimer and collagen-containing extracellular matrix were among the most significant 20 terms. These findings suggest that new cuticle formation and a large amount of energy metabolism may be a crucial adaptive method for *T. spiralis* larval molting. Interestingly, among the structural constituents of cuticle, we found that all upregulated proteins were collagens. Two proteins (E5RZT0 and E5SQP6) were upregulated at IIL compared with ML. At the same time, many cuticle collagen proteins (E5SQP9, A0A0V1BMR6, E5SR67, E5SAG9, A0A0V1BL19 and E5S488) were expressed only at the 10 h IIL stage.

### Kyoto Encyclopedia of Genes and Genome (KEGG) annotation

**Figure 6:**
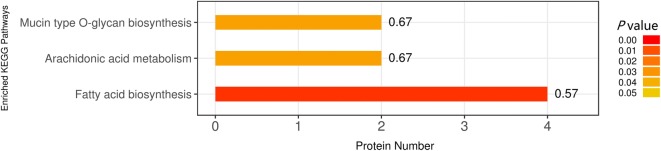
**The most enriched KEGG pathways of differentially expressed** ***T. spiralis*** **proteins between ML and 10 h IIL larvae.** The number denotes the number of proteins involved in the related pathways. The *P* value was calculated by Fisher’s exact test.

**Figure 7:**
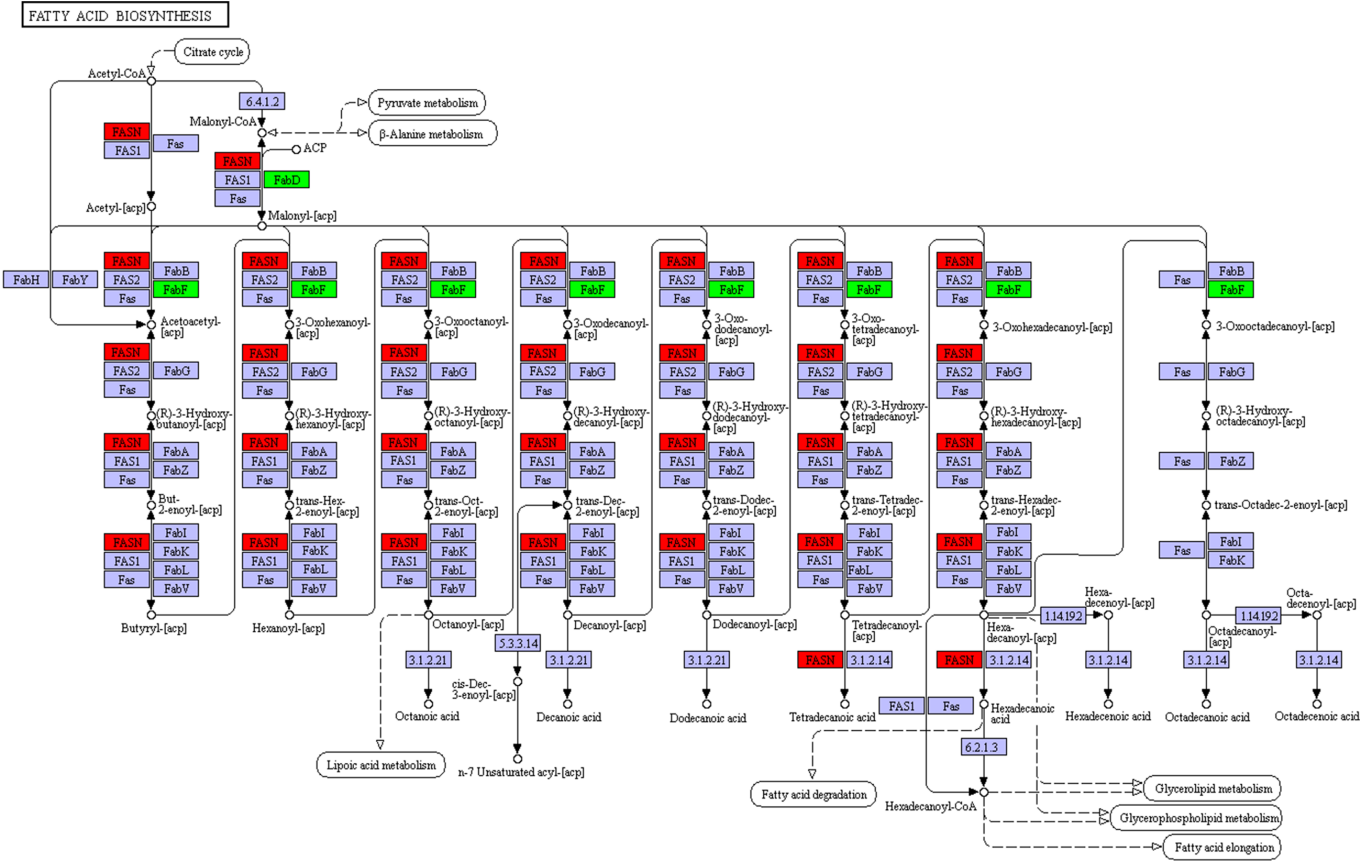
**Representative KEGG pathway for** ***T. spiralis*** **fatty acid biosynthesis.** Four differentially expressed proteins of ML and 10 h IIL were involved in the fatty acid biosynthesis pathway. Red and green symbols represent proteins that were up- or downregulated in *T. spiralis*, respectively.

The biological functions of these 323 differentially expressed *T. spiralis* proteins was further analyzed with the KEGG database, which mapped them to a total of 160 pathways. The most enriched 3 pathways are shown in Figure 6, including fatty acid biosynthesis, arachidonic acid metabolism and mucin type *O*-glycan biosynthesis. The pathway map of fatty acid biosynthesis is presented in Figure 7, which consists of several relevant proteins: fatty acid synthase (A0A0V1BWJ4, E5SGN7), mitochondrial malonyl-CoA-acyl carrier protein transacylase (A0A0V1BUL1) and 3-oxoacyl-[acyl-carrier-protein] synthase (E5SA15). Two fatty acid synthase proteins were upregulated, and the other two proteins were downregulated.

### Protein interaction analysis

**Figure 8:**
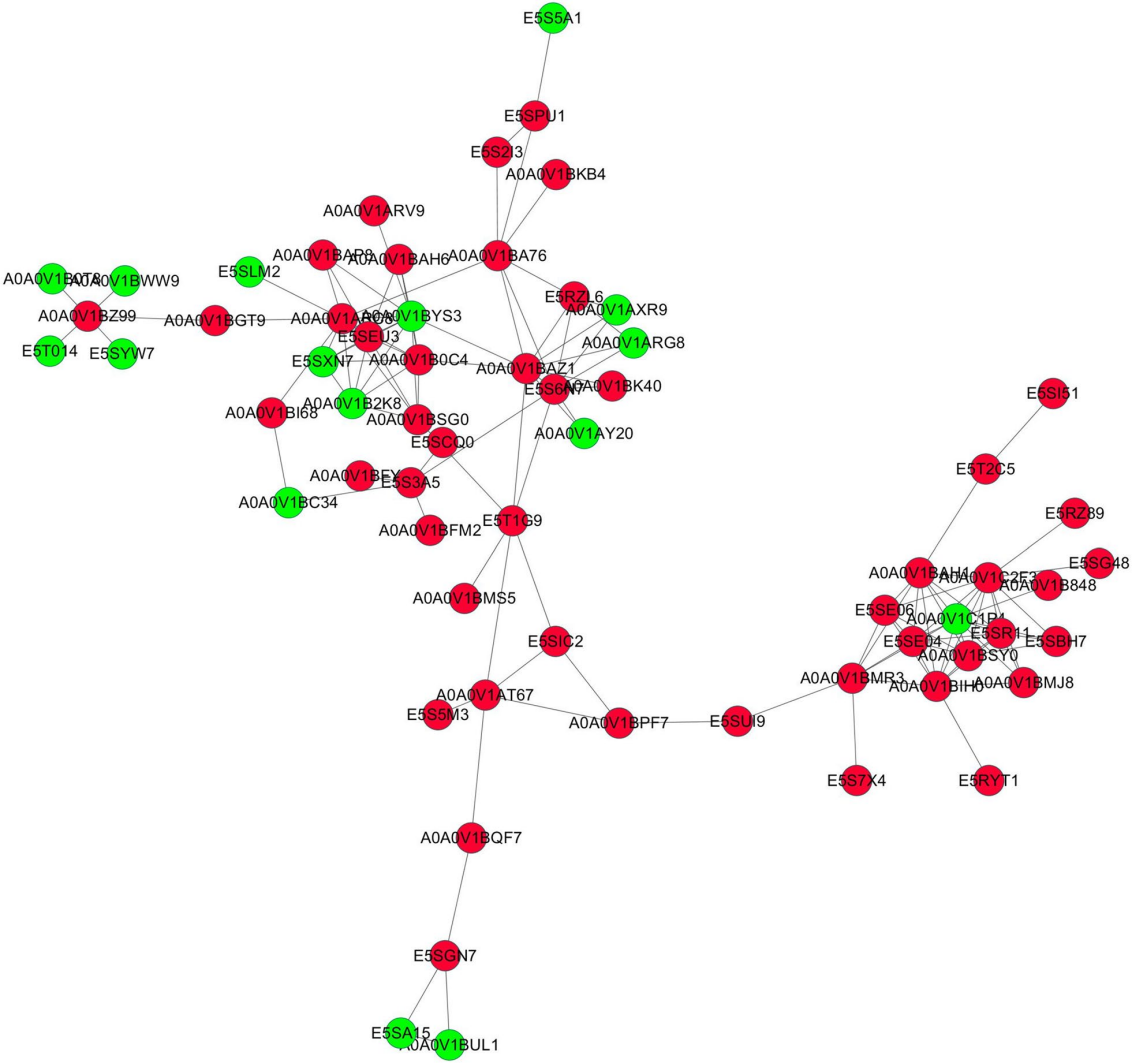
**Protein interaction analysis of differentially expressed** ***T. spiralis*** **proteins between ML and 10 h IIL larvae.** The upregulated (red) and downregulated (green) proteins are marked as nodes.

Network analyses of differentially expressed *T. spiralis* protein were conducted using the STRING database. These differential proteins were associated with each other to generate a network (Figure 8). Proteins situated in the central area were closely connected with larval molting, and the proteins in the core node had high values. In comparison, proteins such as uncharacterized protein (A0A0V1C2F3), ribosome biogenesis protein WDR12-like protein (A0A0V1ARC8), angiotensin-converting enzyme (A0A0V1C1P4), WD repeat-containing protein 3 (A0A0V1BYS3), 28S ribosomal protein S11, mitochondrial (A0A0V1BAZ1) and uncharacterized protein (A0A0V1BIH0) were situated in the connected nodes of the protein functional interaction network. These proteins are involved in intrinsic components of membranes, metallopeptidase activity, ribosome biogenesis, and serine-type endopeptidase inhibitor activity.

### qPCR validation of differentially expressed *T. spiralis* genes

**Figure 9:**
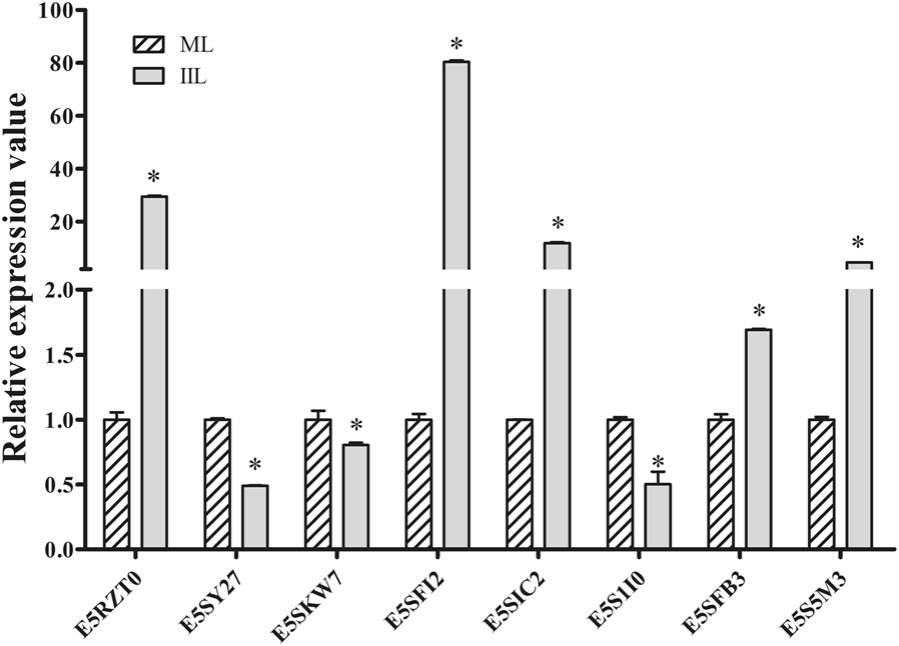
**qPCR validation of differentially expressed** ***T. spiralis*** **genes.** Comparison of the relative quantitation of each gene expressed in ML and 10 h IIL. The transcriptional levels of all eight *T. spiralis* genes were significantly different in the two stages of worms (*P *< 0.05).

To further assess the differential expression of the molting-related proteins in ML and 10 h IIL, the transcription levels of eight genes, which we chose based on interest and their different ratios, were determined via qPCR, and the results are shown in Figure 9. Out of these eight *T. spiralis* genes, five (cuticle collagen 14, putative DOMON domain-containing protein, glutamine synthetase, cathepsin F and NADP-dependent isocitrate dehydrogenase) had a significantly higher transcriptional levels in 10 h IIL than that at the ML stage (*P *< 0.05), which were similar to their protein expression levels. However, the transcription levels of three genes (cytochrome P450 4V2, endoplasmic oxidoreductin-1 and RNA-binding protein 47) showed low compliance with their protein expression levels.

## Discussion

*Trichinella* infection begins when infective muscle larvae are ingested. This larval phase has been used as the starting time for investigating larval ecdysis and the development of this nematode. Muscle larvae maintain a relatively stable state in the intracellular microenvironment. Once they enter the intestinal epithelium niche, four molts ensue that the larvae quickly grow to adulthood, within 31 h [7]. The selective pressures favor the larvae’s completing the ecdysis during such a short time. However, the first molting times of the IIL larvae vary from 6 to 12 h [16]. The difference in larval molting time might be related to different hosts or/and isolates. Therefore, we observed the molting times of *T. spiralis* IIL using an individual isolate (ISS534) in the same host (BALB/c mice). Our results revealed that IIL began the first molting at 10 hpi, which was consistent with the results of others [16]. Hence, we selected ML and 10 h IIL to investigate their differentially expressed proteins.

*Trichinella* larvae might require a physical barrier against which to rub for removing their outer cuticle layer and need a complex regulatory system to complete the sloughing of old cuticle and generation of new cuticle. Although complete larval ecdysis has been observed while the larvae were cultured in vitro, the molting mechanism was not clarified in as much detail as, for instance, in certain free-living nematodes such as *Caenorhabditis elegans* [12, 13, 31]. Advances in proteomics, genomics and bioinformatics techniques have provided a feasible way to study the molecular basis of nematode larval ecdysis and development [32]. In the present work, proteomic profiles of *T. spiralis* ML and 10 h IIL were analyzed with LC–MS/MS to identify the molting-related proteins. A total of 2885 proteins of *T. spiralis* were identified, 323 of which were differentially expressed.

Our results showed that some peptidases were upregulated in 10 h IIL, including cathepsin (E5SFB3, S5M797), metalloprotease (E5S4Y1, A0A0V1C237), and carboxypeptidase (E5SP83). However, we did not know whether these peptidases were molting-related enzymes. Previous studies showed that during the development of L3 to L4 larvae of lymphatic filarial *Brugia pahangi*, larval molting depended on the cathepsin L enzymatic activity, which was identified in excreted/secreted proteins of molting L3 larvae [33]. In *C. elegans*, an elevation of cathepsin L transcription level was observed in the intermolt period approximately 4 h before each molting. RNA interference with the enzymatic activity of cathepsin L brought about embryonic fatality and a prolonged development of larvae to adulthood, demonstrating a major function of this protease for embryogenesis and larva growth [34]. A previous study indicated that cathepsin L and Z were indispensable for ecdysis of *Onchocerca volvulus* L3–L4 larvae by immune-electron microscopy [35]. Similar to cathepsin, metalloproteases play an important role in cuticle ecdysis. The *C. elegans* nas-36 and nas-37 genes encode astacin metalloproteases. When they are mutated, larva molting defects result, e.g., the incapacity of the larvae to shed previous old cuticle [36]. In addition, carboxypeptidase is necessary for new cuticle generation and body morphogenesis in free-living and parasitic nematodes, and it is located at the hypodermis and interacts with other collagen-modifying enzymes [37]. Therefore, hydrolases play an important role in the molting process.

There are other types of proteases that play important regulatory roles in molting, such as E3 ligase (A0A0V1B0D3) and serine/threonine-protein kinase RIO1 (A0A0V1BAH6). Similarly, our results indicated that the expression of these two proteases was elevated in the 10 h IIL. In *C. elegans*, the expression level of E3-ligase RNF-5 is raised, especially during larval molting, and this enzyme ubiquitinates the dense body protein so that the old cuticle sheds [38]. NEKs are serine-threonine protein kinases that participate in cellular mitosis. Using a genetic approach, a previous study revealed that NEKL-3 was indispensable for completing larval molting. A hypomorphic mutation of NEKL-3 and sv3 in *C. elegans* resulted in the inability of larvae to escape from the old cuticle. Inhibition of NEKL-2 produced the mislocalization of leucine-responsive regulatory protein-1 (LRP-1)/megalin, a cell surface receptor of low-density lipoprotein binding protein [39]. The LRP mutations of *C. elegans* resulted in an obvious defect, an inability to remove all old cuticles at each larval molting stage [40]. Genetic mosaic analysis suggests that the LRP-1 gene plays a role in the major epidermal syncytium hyp7, a polarized epithelium that secretes cuticle from its apical surface. Consistent with this, NEKL-2 and NEKL-3 are expressed at the apical surface of epidermal syncytium hyp7 [39, 40]. In our study, the expression of *T. spiralis* low-density lipoprotein receptor-related protein (A0A0V1AUS8) was consistent with serine/threonine-protein kinase.

In a GO enrichment analysis, the structural constituent of the IIL cuticle was the major category of molecular function. We found that some collagen proteins (E5RZT0, E5SQP6) were upregulated at 10 h IIL, and many cuticle collagen proteins (E5SQP9, A0A0V1BMR6, E5SR67, E5SAG9, A0A0V1BL19 and E5S488) were expressed only in 10 h IIL. Collagen is a structural protein of extracellular matrices. Nematodes can alter cuticle surface protein components between two molting stages or when the ambient environment changes; surface protein alteration can allow parasitic nematodes to escape from the host’s immune defenses in the process of infection [41]. Previous studies revealed that schistosome tegument plays an important role in *Schistosoma* infection, such as by promoting juvenile worm growth, nutrition and immune evasion and modulation [42]. Other studies have indicated that the *C. elegans* cuticle consists of a complex collagen matrix. A single gene mutation of cuticle collagen could result in a cuticle defect that changes the nematode’s morphology [43]. Nuclear hormone receptors (NHRs) play a vital role in collagen synthesis. A putative nuclear hormone receptor, NHR3 (A0A0V1AYJ5), was expressed only at the 10 h IIL stage. In *C. elegans*, CHR3 is crucial for cuticle formation and molting, while the NHR-23 expression and decreased NHR3 function during the intermolt time lead to a subsequent molt defect [44, 45]. Carboxylic acid binding proteins (A0A0V1B0P6, A0A0V1B0I4 and A0A0V1B0M0) occupied the greatest proportion of binding activity proteins, and all these proteins were up-regulated in 10 h IIL. However, the function of these proteins in the process of molting is unclear and needs to be further studied. *T. spiralis* undergoes a process of molting 4 times, which involves an interplay between various intracellular pathways. KEGG pathway analysis indicated that differentially expressed proteins were involved in three signaling pathways: fatty acid biosynthesis, mucin type *O*-glycan biosynthesis and arachidonic acid metabolism. Knockdown of the genes that encode fatty acid biosynthesis proteins with RNAi resulted in serious defects in triglyceride production and *C. elegans* larval molting*s*. Downregulation of these protein’s gene expression impaired the new cuticle generation and destroyed the larval cuticle integrity [46]. Other studies demonstrated that a lipoxygenase pathway product is necessary for ecdysis of infective larvae of filarial parasites. When inhibitors of arachidonate metabolism were added to in vitro cultures containing *B. malayi* L3, they were capable of preventing larval development [47]. The relationship between mucin type *O*-glycan biosynthesis and ecdysis is unclear. *Trypanosoma cruzi* is capped with a dense coat of mucin-type glycoproteins, which is vital to facilitate this protozoon’s intrusion and parasitism in the host cell [48]. Our results also showed that the protein interaction network center contained ribosome biogenesis protein WDR12-like protein (A0A0V1ARC8), Sel1 repeat family protein (E5S6N7), U3 small nucleolar RNA-interacting protein 2 (A0A0V1B0C4), 28S ribosomal protein, and mitochondrial (S11A0A0V1BAZ1). These proteins might be key for ecdysis and the development of IIL.

In this work, the qPCR results demonstrated that the transcription levels of five *T. spiralis* genes (cuticle collagen 14, putative DOMON domain-containing protein, glutamine synthetase, cathepsin F, and NADP-dependent isocitrate dehydrogenase) were significantly higher at the IIL stage than at the ML stage, which was consistent with the quantitative proteomic analysis results. However, the protein and mRNA levels of the other three genes (cytochrome P450 4V2, endoplasmic oxidoreductin-1, RNA-binding protein 47) were not consistent. The contradiction might be due to post-translational control, in which negative regulatory factors are possibly activated in the translation process, or the intrinsic mRNA is regulated by specific molecules such as microRNA [23]. The proteomic information obtained from this study is important for *T. spiralis* molting and development. These proteins are comparable to the homologous proteins of other nematodes and might be valuable as molecular targets to exploit vaccines and new drugs against this parasitic nematode. Furthermore, studies on molting-related protein function will further clarify the mechanism and control of molting in parasitic nematodes [31].

Our results are the first to establish extensive proteomic information on the molting larvae of *T. spiralis*. A total of 323 differentially expressed *T. spiralis* proteins were identified by LC–MS/MS analysis combined with bioinformatics, and their molecular functions (binding, catalytic and transporter activity) were annotated. Some proteins are primarily involved in cuticle structural elements, cuticle synthesis, maintenance, remodeling and degradation, hormonal regulation of molting and intracellular trafficking components and regulators. Five *T. spiralis* genes (cuticle collagen 14, putative DOMON domain-containing protein, glutamine synthetase, cathepsin F and NADP-dependent isocitrate dehydrogenase) had significantly higher transcriptional levels at the 10 h IIL than at the ML stage (*P* < 0.05), which were similar to their protein expression levels, suggesting that they might be *T. spiralis* molting-related genes. Further understanding of the regulatory mechanism of larval ecdysis and the function of ecdysis-related proteins will offer an important foundation to develop new vaccines and drugs against *T. spiralis* infection.

## Abbreviations

AW: adult worm
GO: gene Ontology
hpi: hours post-infection
ILL: intestinal infective larvae
KEGG: Kyoto Encyclopedia of Genes and Genomes
LRP-1: leucine-responsive regulatory protein-1
ML: muscle larvae
SD: standard deviation
SPF: specific-pathogen-free
